# Longitudinal Performance in Basic Numerical Skills Mediates the Relationship Between Socio-Economic Status and Mathematics Anxiety: Evidence From Chile

**DOI:** 10.3389/fpsyg.2020.611395

**Published:** 2021-01-14

**Authors:** Bárbara Guzmán, Cristina Rodríguez, Roberto A. Ferreira

**Affiliations:** ^1^Facultad de Educación, Universidad Católica de la Santísima Concepción, Concepción, Chile; ^2^Facultad de Psicología, Universidad de la Laguna, Tenerife, Spain; ^3^Facultad de Educación, Pontificia Universidad Católica de Chile, Santiago, Chile

**Keywords:** mathematics anxiety, socio-economic status, basic numerical skills, mathematical performance, mediation

## Abstract

Socio-economic status (SES) and mathematical performance seem to be risk factors of mathematics anxiety (MA) in both children and adults. However, there is little evidence about how exactly these three constructs are related, especially during early stages of mathematical learning. In the present study, we assessed longitudinal performance in symbolic and non-symbolic basic numerical skills in pre-school and second grade students, as well as MA in second grade students. Participants were 451 children (average pre-school age = 5 years, 6 months) from 12 schools in Chile, which differed in school vulnerability index (SVI), an indicator of SES. We tested an explanatory model of MA that included SES and longitudinal performance in basic numerical skills as predictors. The results showed a direct effect of SES on MA and a mediating effect of performance in symbolic and non-symbolic comparison tasks in pre-school. However, in second grade, only performance in symbolic comparison significantly mediated the SES-MA relationship. These findings suggest that performance in non-symbolic comparison plays an important role in explaining MA at initial stages, but that its influence is no longer significant by the time children reach formal instruction in second grade. By contrast, as children’s formal educational experience in mathematics increases, MA becomes linked primarily to symbolic numerical tasks. In sum, SES affects MA and this is due in part to the effect of SES on the development of numerical learning in pre-school, which in turn has an impact on subsequent, more complex learning, ultimately leading to differences in MA. We discuss the implications of these findings for preventing and acting upon the emergence of MA.

## Introduction

Mathematics anxiety (MA) is an unpleasant emotional response to numerical and arithmetic tasks that interferes with mathematical performance (Richardson and Suinn, 1972; Ashcraft and Faust, 1994; Dowker et al., 2016). To date, most of the evidence for MA has been gathered from adult participants, but in recent years it has been discovered that primary school children also report feelings of stress and fear associated with mathematics (Devine et al., 2012; Wu et al., 2012; Young et al., 2012; Ramirez et al., 2016). Although a clear link between mathematical performance and MA has been established, the reasons for the emergence of MA and the precise developmental point at which it occurs are not entirely clear.

It is possible that the factors underlying MA come from individuals themselves (i.e., emotional reaction and cognitive performance) and from the environment (Chang and Beilock, 2016; Rubinsten et al., 2018). Among the individual-level factors, mathematical performance is the most widely studied. However, the mathematical performance-MA relationship is complex. Theoretically, two explanatory models of this relationship have been proposed. The Deficit Theory states that early low performance elicits MA (Tobias, 1986; Maloney et al., 2011), so students with low math skills experience difficulties in their learning and are more exposed to developing negative emotions. By contrast, the Debilitating Anxiety Model posits that MA negatively affects performance through two possible pathways. First, the intrusive thoughts and worries associated with MA use cognitive resources such as working memory, which are needed to solve mathematical tasks (Caviola et al., 2012; Ching, 2017). Second, students with MA show an avoidance effect toward mathematical learning. For example, under time pressure in evaluation contexts, they trade off speed for accuracy (Ashcraft and Faust, 1994). Both theories are supported by convincing evidence gathered over time; however, recently it has been suggested that the relationship between MA and math performance is likely to be bidirectional. The Reciprocal Theory (see Carey et al., 2016, or Foley et al., 2017 for review) combine both explanations. Under this approach, MA and mathematical performance work as a vicious feedback loop. That is, poor math performance leads to MA and later on MA, in turn, leads to poor math performance. Research regarding environmental factors is somewhat scarce, but the data available suggests that socio-economic status (SES) might play a significant role in MA. For instance, data from the Program for International Student Assessment (PISA) indicate that, on average, countries with low SES show high MA rates, whereas economically developed countries report low MA levels (Organisation for Economic Co-operation and Development, 2013; Stoet et al., 2016; Foley et al., 2017). It has been suggested, however, that the link between SES and MA is more complex, since SES has also been shown to affect mathematical performance (Mazzocco et al., 2011), and MA and mathematical performance are strongly related. A very good example of the complex relationship among SES, MA, and mathematical performance are Latin American OECD participant countries. Students from these countries show the highest MA levels and very low mathematical achievement. Chile, in particular, is the country with the highest MA rates among secondary school students, ranked only below Mexico (Organisation for Economic Co-operation and Development, 2013). Thus, studying the SES-MA-performance relationship in Chile is of high interest, especially in children at initial stages of their education, and considering that Chile’s education system is highly stratified by school SES.

### Relationship Between Mathematics Anxiety and Mathematical Performance

The directionality of the relationship between MA and mathematical performance is still under debate. Both the Deficit Theory and the Debilitating Anxiety Model have received empirical support. The recent review by Carey et al. (2016) has brought more consensus to the field, suggesting that MA and mathematical performance are more likely to influence each other in a vicious circle that probably starts with poor math performance, giving rise to MA and, over time, MA would also influence mathematical performance. In order to better understand the complex interplay between MA and mathematical performance, it is important to examine findings from longitudinal studies.

Up to date, there are very few longitudinal studies looking at MA and math achievement, and their findings suggest that early math achievement is more likely to influence MA than vice versa (see Cargnelutti et al., 2017; Gunderson et al., 2018; Sorvo et al., 2019). Cargnelutti et al. (2017) followed children from second to third grade and tested their mathematical performance (average scores of written computation, word problems, and the MAT-2 module Number) and MA. The authors found that MA had a stronger overall predictive role on mathematical performance than vice versa. However, a more careful inspection of the longitudinal analysis revealed that MA and mathematical performance actually influenced each other, with mathematical performance influencing MA in second grade, and MA influencing performance in third grade. The authors suggested that poor performance at early stages can trigger MA, but later on, it is MA that mostly affects performance. In a more recent study, Gunderson et al. (2018) also found a reciprocal relationship between MA and mathematical achievement in first and second grade students using word problems involving simple arithmetic, monetary calculations, and measurement. It is worth noting, however, that the impact of early mathematical achievement on later MA was much stronger than the effect of early MA on later mathematical achievement. A third study that has shed light on the relationship between MA and mathematical achievement is that of Sorvo et al. (2019). Over a period of a year they studied MA and mathematical achievement in second and fifth grade students and discovered that low arithmetic achievement predicted later MA. The findings from these three longitudinal studies coincide regarding the existence of a clear and stable relationship between MA and mathematical achievement over time. While two of the studies agree that the relationship is reciprocal (Cargnelutti et al., 2017; Gunderson et al., 2018), there is also agreement that early mathematical achievement is more likely to influence MA later on than vice versa. These findings once again confirm the reciprocal relationship between mathematical performance and MA, in line with the Reciprocal Hypothesis (Carey et al., 2016). Additionally, they suggest that mathematical learning could be at the start of the vicious cycle, partially supporting the Deficit Theory (Tobias, 1986; Maloney et al., 2011).

Research supporting the Deficit Theory suggests that a deficit at a basic level (i.e., less precise understanding of numerical magnitudes) could underlie the mathematical performance-MA relationship (Tobias, 1986; Maloney et al., 2010; Núñez-Peña and Suárez-Pellicioni, 2014). This deficit in basic numerical skills could later affect the development of more complex mathematical learning. Data from university students have shown that high-MA individuals perform worse than their low-MA peers in basic numerical processing tasks such as visual enumeration, number comparison, and symbolic magnitudes (Maloney et al., 2011; Núñez-Peña and Suárez-Pellicioni, 2014). In line with the above results, more recent studies have demonstrated that high-MA individuals also have a poorer approximate number system or more limited pre-verbal numerical representation than their low-MA peers, supporting the cognitive deficit model (Lindskog et al., 2017). Other studies, however, display no relationship between MA and mathematical performance in basic numerical tasks such as dot comparison, numerical comparison, and counting (Dietrich et al., 2015; Douglas and LeFevre, 2018; Colomé, 2019). In children, very few studies have analyzed the association between MA and basic numerical performance and, to the best to our knowledge, none in early stages of mathematical learning (Wang et al., 2015; Hart et al., 2016). Hart et al. (2016) studied the profiles of mathematics performance in children (Age, *M* = 12.25, *SD* = 1.20). They used an MA questionnaire, two basic numerical tasks (dots estimation task and number line estimation task) and two standardized math achievement tasks (calculation and applied problems), among other tasks. Six classes emerged from the analysis, but only two were characterized by high levels of MA, both presented low math achievements and only one also showed low performance in number line estimation. None of the classes showed high MA levels and low performance in dots estimation.

### Relationship Between Socio-Economic Status and Mathematics Anxiety

Since MA is a complex construct, the factors that contribute to its emergence and development may go beyond intrinsic individual characteristics. According to PISA 2012, significant differences in MA levels were reported by secondary school students as a function of their SES (Organisation for Economic Co-operation and Development, 2013). Low-SES students show significantly more MA than their higher-SES peers. The empirical evidence, however, is not so clear-cut. For instance, a study of university students in Turkey found that SES, measured according to family income and parents’ educational attainment, negatively affected MA levels reported by students (Geyik, 2015). However, a study of secondary school students in India found no relationship between parents’ income and student MA levels (Mahigir et al., 2012).

As is the tendency across all factors relating to MA, the evidence linking SES and MA in younger populations is much more limited than that available for secondary and higher education. Findings obtained from primary school children show similar discrepancies to those from secondary and university students, presented above. For example, a cross-sectional study involving children aged 6 to 12 years in Nigeria showed that MA levels differed significantly according to SES (measured in terms of parental occupation, income, and educational attainment), with anxiety being prevalent among low-SES students (Adimora et al., 2015). By contrast, in a recent longitudinal study investigating the relationship between MA and mathematical performance in Chinese children from second to third grade, no significant influence of SES (defined as the educational attainment of the mother) over MA was found (Ching, 2017). However, the striking differences between these two studies in terms of how SES is determined are likely to explain the inconsistent findings. In general, it is advisable to include composite measures of SES in order to ensure a more representative estimation (Letourneau et al., 2011), as was the case with Adimora et al. (2015). Hence, there is a need for new studies that follow these guidelines and present data from a wider range of countries, where SES could potentially have an influence on MA and/or mathematical achievement.

In Chile, more than half of primary school children in the fourth and eighth grades assessed by the Chilean national curriculum assessment system (SIMCE) report feeling anxious about subjects that include mathematical content (Agencia de Calidad de la Educación, 2017). More importantly, MA levels among socio-economically disadvantaged students are significantly higher (*M* = 0.52, *SD* = 0.002) than those of students from more privileged backgrounds (*M* = 0.28, *SD* = 0.03). Although the evidence is not conclusive in other parts of the world, in Chile the data reported by children in fourth grade and above are consistent in showing a negative relationship between SES and MA (Agencia de Calidad de la Educación, 2017). However, up to date, no studies have looked into the SES-MA relationship in younger children either in Chile or elsewhere.

Given the reduced number of studies that have addressed the SES-MA relationship, a theoretical framework to discuss this phenomenon is very limited. Some have argued that low-SES is related to mental and health problems, resulting from disruptions in parenting and family conflicts (Adimora et al., 2015), which would explain the differences in MA between low and high-SES children. Others have noticed that high-SES results in high parent educational level (Geyik, 2015), which has an impact in how children regulate their emotions toward subject disciplines. In particular, the author suggests that parents, who attain high educational level, are also well educated about developing positive feelings about mathematics, which would be transmitted to their children. A third underlying factor that may explain the relationship between SES and MA is stereotype threat–the case in which members of a group or community are afraid or feel at risk of confirming a stereotype about their group. In this framework, MA could be the result of an emotional reaction of children from low-SES backgrounds triggered by the stereotype threat on mathematics (Carey et al., 2016). If low-SES children are aware of the negative stigma regarding their mathematical abilities, they could develop a negative attitude and unpleasant emotions toward school and mathematical learning.

### The Present Study

In the present investigation, we tested an explanatory model of MA that includes SES and longitudinal performance in basic numerical skills as predictors. As discussed earlier, a direct relationship between SES and MA has been established in secondary and university students. However, it is not clear whether this link is also present at early stages of formal instruction and whether mathematical achievement plays a mediating role in the relationship. Therefore, in the present study, we assessed whether performance in basic numerical skills in pre-school and second grade mediates the relationship between SES and MA. We study the longitudinal effect of basic numerical skills based on evidence that the effect of SES and MA on mathematical performance differs over time (Jordan et al., 2010; Sorvo et al., 2019).

We hypothesize that longitudinal performance in basic numerical skills mediates the relationship between SES and MA. To study basic numerical skills, numerical magnitude processing measures were included because of their ability to predict subsequent performance in mathematics (Nosworthy et al., 2013; Link et al., 2014). We also used symbolic and non-symbolic magnitude comparison tasks in order to identify early deficits in mathematics (Siegler and Booth, 2004; De Smedt et al., 2013) which, according to the cognitive deficit model, could be related to the emergence of MA later. We expect to find a direct effect of SES on MA in line with studies carried out in adolescents (Organisation for Economic Co-operation and Development, 2013; Geyik, 2015), as well as a mediating effect of simple numerical skills between SES and MA, given previous evidence in adults (Lindskog et al., 2017; Douglas and LeFevre, 2018).

## Materials and Methods

### Participants

Participants were 613 children (Time 1) and 486 (Time 2). After data collection, 35 students were excluded due to missing data in one of the measurements. Hence, the final sample of students was 451 children (225 girls) (mean pre-school age = 5 years, 6 months). All participants were typically developing children with no special educational needs, intellectual disabilities, or neurological disorders.

The Chilean school system has three types of schools that differ in their funding mechanisms. Public schools receive a government subsidy, private-subsidized schools receive state funding and private grants from families, and private schools only receive grants from family. In the present study, the students were drawn from 12 urban schools (three public, seven private-subsidized, and two private) from a southern region of Chile. The Chilean educational legislation assigns each school a school vulnerability index (SVI) that is published annually by the National Board of School Aid and Scholarships (JUNAEB) (Ministerio de Educación de Chile, 2019) (see “Measures” section). We used the SVI (*M* = 53.4, *SD* = 23.5) of the 12 schools involved in the study as SES index. See Table 1 for descriptive statistics.

**TABLE 1:** Number of students by school and gender.

School	SVI/SES	*N*	Students by gender	
			Girls	Boys
1	0.00	14	17	19
2	0.00	22	10	7
3	32.22	39	29	25
4	39.97	50	19	20
5	45.66	60	8	6
6	47.53	37	9	13
7	52.63	20	29	31
8	54.07	59	28	31
9	77.19	43	10	10
10	77.32	36	26	24
11	82.16	54	13	30
12	91.80	17	27	10
Total		451	225	226

### Measures

#### School Vulnerability Index (SVI)

The SVI is obtained from a vulnerability survey administered to each family and comprises schooling and occupation of the parents, characteristics of the student’s home, family context, and social relationships, students’ study habits, among others. In addition, the SVI considers complementary information on the use of State aid for the protection and social reintegration of children and adolescents, as well as for overcoming poverty. For instance, The National Health Fund (FONASA) provides information about health quality of the student and their family; The Chilean Civil Registry provides identification data for the family (number of children, marital status, assets, etc.); the Ministry of Education (MINEDUC) provides information regarding students’ grades and class attendance.

School vulnerability index is calculated by rating students within three poverty levels. The first level brings together students at the highest socioeconomic risks; the second level include students with less socioeconomic vulnerability than the above, but that present socio-educational risks associated with low school performance and attendance, or dropout from the educational system. The third level gathers students with a socioeconomic vulnerability similar to that of the second level, but without socio-educational risk. The index is obtained by weighting the proportion of students belonging to the three poverty levels in each school, and ranges from 0 to 100, with higher numbers indicating greater vulnerability.

#### Mathematics Anxiety

The Spanish Adaptation of the Child Mathematics Anxiety Questionnaire-Revised (CMAQ-R) (Ramirez et al., 2016) was used (see Guzmán et al., 2020). The instrument was administered on an individual basis to first and second grade children. It covers 16 situations involving mathematics, half of which constitute specific mathematical problems and the other half are mathematical situations in the classroom. Using a scale of five faces that express emotional states from no anxiety to very, very anxious, the children were required to indicate the face which expresses the level of anxiety that they feel in each of the proposed situations. The total MA score is calculated based on the total responses of each item in a range of 16 to 80 points. The Chronbach’s Alpha for both the original test and the Spanish adaptation is 0.83, and 0.81 for the current sample.

#### Basic Numerical Skills

Two numerical tasks were applied, one in pre-school and one in second grade. These have been widely used to evaluate numerical performance in formal pre-school and early primary school education (Clarke et al., 2008; Nosworthy et al., 2013). Both measures were applied on an individual basis and with a time limit of a minute.

##### Cross-out symbolic comparison task (S_C)

The task consisted of a booklet containing 75 pairs of one- and two-digit numbers. The difficulty of the stimuli was manipulated by varying the ratio between the numbers, so task difficulty increased as the ratio approached 1. There were 25 low difficulty pairs (ratios between 0.11 and 0.36), 25 medium difficulty pairs (ratios between 0.37 and 0.62), and 25 high difficulty pairs (ratios between 0.63 and 0.89). Children were given a minute to cross out as may higher numbers as possible. The stimuli were presented randomly, and the position of the largest digit was counterbalanced. Four examples were given before the task. The number of correct responses was converted into a final score. Based on a randomized subsample of 150 children, internal consistency was 0.89 and 0.90 in pre-school and second grade, respectively.

##### Cross-out non-symbolic comparison task (NS_C)

The task consisted of a booklet containing 75 pairs of rectangles filled with groups of black dots. Stimuli were created using Panamath (Halberda et al., 2008). Each group had between 1 and 20 dots, and difficulty was manipulated by varying the ratio between dot arrays (25 low difficulty pairs, ratios between 0.25 and 0.41; 25 medium difficulty pairs, ratios between 0.42 and 0.70; 25 high difficulty pairs, ratios between 0.71 and 0.90) and dot size (25 pairs not size controlled; 25 pairs size controlled, where the total area of one group of dots was equal to that of the other group; and 25 pairs anti-correlated, where the total area of one group of dots was double that of the other group). Children were given a minute to cross out the group with the higher number of dots in as many pairs as possible. Two examples were given before the task. The number of correct responses was converted into a final score. Based on a randomized subsample of 150 children, internal consistency was 0.77 and 0.71 in pre-school and second grade, respectively.

### Procedure

The numerical tasks were administered on an individual basis in pre-school (T1) and second grade (T2) by trained examiners. The CMAQ-R questionnaire was administered only in second grade. Numerical tasks and CMAQ-R were administered in two separate sessions to avoid possible interference which might affect the results. The study received prior approval from the Ethics Committee of the Universidad Católica de la Santísima Concepción, and consent and assent forms were sent to parents for signature prior to the commencement of the study.

### Data Analysis

Missing data were handled using complete-case (CC) analysis, so no imputation or replacement of missing data was performed. The CC approach was used because of its validity to work with missing completely at random (MCAR) data (White and Carlin, 2010; Karahalios et al., 2012; van Ginkel et al., 2020), as this was the case in the present study^1^. Two phases of analysis were conducted: (1) bivariate correlations to evaluate associations between all of the variables included in the main analyses; (2) serial mediation analysis to determine whether the capacity to compare symbolic and non-symbolic magnitudes in pre-school and second grade mediate the SES-MA relationship. Serial mediation models were calculated using model 6 from the PROCESS v.2.15 macro. The analysis was based on 5,000 bootstrap iterations, and the confidence interval (CI) was set to 95%, as recommended by Preacher et al. (2007). When zero is not included in the 95% confidence interval, the indirect effect of the independent variable (i.e., SES) on the dependent variable (i.e., mathematics anxiety) is mediated by the proposed sequential mediators (i.e., basic numerical skills) (Hayes, 2018). Bootstrapping test of mediation was used because it provides increased power over traditional tests of mediation and does not assume a normal distribution to the data (Hayes, 2018). Effect size in mediation analysis was assessed with completely standardized indirect effect size (effect size_*cs*_). According to Preacher and Kelley (2011), the effect size_*cs*_ is interpreted as small (0.01), medium (0.09), and large (0.25).

## Results

### Descriptive Statistics and Correlations of Variables

Descriptive statistics and correlations between the variables are shown in Table 2. MA score was correlated negatively and significantly with all of the numerical variables, regardless of age group and task type, with the exception of SES, which was positively correlated with MA. SES was correlated significantly with all of the numerical tasks, with the exception of the non-symbolic comparison tasks in second grade (T2_NS_C). All of the numerical variables were positively correlated among themselves.

**TABLE 2:** Pearson correlations between all measured variables and descriptive statistics for all measured variables (*N* = 451).

Descriptive statistics					Correlations					
Measures	M	SD	Skew	Kurtosis	1	2	3	4	5	6
1. SES	50.99	26.03	–0.41	–0.64		0.25**	−0.27**	–0.21^∗∗^	–0.24^∗∗^	0.00
2. MA	41.16	11.90	0.09	–0.28			−0.29**	–0.21^∗∗^	–0.26^∗∗^	−0.12^∗^
3. T1_S_C	16.56	9.48	0.06	–0.96				0.47^∗∗^	0.48^∗∗^	0.13^∗∗^
4. T1_NS_C	19.54	6.20	0.25	0.73					0.31^∗∗^	0.29^∗∗^
5. T2_S_C	25.65	10.14	–0.44	0.14						0.22^∗∗^
6. T2_NS_C	24.67	7.48	0.22	0.61						

T1_S_C = symbolic comparison in pre-school; T1_NS_C = non-symbolic comparison in pre-school; T2_S_C = symbolic comparison in second grade; T2_NS_C = non-symbolic comparison in second grade. * *p* < 0.05. ** *p* < 0.01.

### Serial Mediation

#### Model 1

**FIGURE 1:**
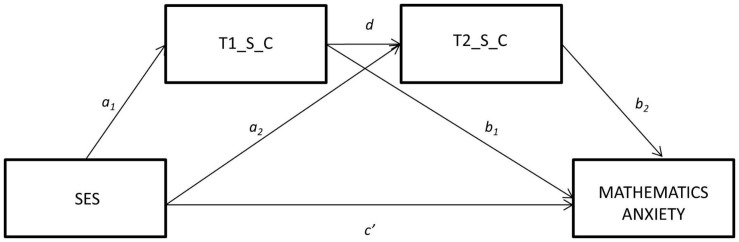
Serial mediation model for the relationship between socio-economic status and mathematics anxiety by longitudinal performance in symbolic numerical skills (Time 1 and Time 2).

The first serial mediation model examined whether the relationship between SES and MA was mediated by longitudinal performance in symbolic numerical skills (Figure 1). Results revealed that the SES-MA relationship was mediated by performance in symbolic magnitude comparison in pre-school (T1_S_C) and second grade (T2_S_C). The total indirect effect was 0.08, which suggests a small to moderate effect size. The specific effects are reported in Table 3. The total effect of SES on MA (combining direct and indirect effects) was significant (*c* = 0.12, *SE* = 0.02, *p* < 0.001), showing that MA is greater in more vulnerable children and those who present poorer mathematical performance in symbolic number comparison tasks. The effect size_*cs*_ for the mediation model was 0.25, which is considered a large effect size. The estimated standardized path coefficients are reported in Table 4.

**TABLE 3:** Indirect effects in the serial mediation models.

	Model 1			Model 2		
Mediator	Effect	BootSE	95% CI	Effect	BootSE	95% CI
Ind 1	0.05	0.02	[0.01, 0.08]	0.03	0.01	[0.01, 0.05]
Ind 2	0.02	0.01	[0.00, 0.03]	–0.01	0.01	[−0.01, 0.00]
Ind 3	0.02	0.01	[0.00, 0.03]	0.01	0.00	[−0.01, 0.01]

*Ind 1 = SES → pre-school → MA; Ind 2 = SES → second grade → MA; Ind 3 = SES → pre-school → second grade → MA.*

**TABLE 4:** Estimated standardized path coefficients by serial mediation models.

	Model 1			Model 2		
Paths	β	*SE*	*t*	β	*SE*	*t*
*a*_1_	–0.11^∗∗∗^	0.02	–5.69	–0.06^∗∗∗^	0.01	–4.34
*a*_2_	–0.05^∗∗^	0.02	–2.62	0.02	0.02	1.38
*b*_1_	–0.22^∗∗∗^	0.07	–3.39	–0.26^∗∗^	0.09	–2.78
*b*_2_	–0.16^∗∗^	0.06	–2.59	–0.14	0.08	–1.77
*d*	0.49^∗∗∗^	0.05	10.34	0.36^∗∗∗^	0.06	6.34
*c*′	0.08^∗∗∗^	0.02	3.58	0.11^∗∗∗^	0.02	4.68
*c*	0.12^∗∗∗^	0.02	5.30	0.12^∗∗∗^	0.02	5.32

**FIGURE 2:**
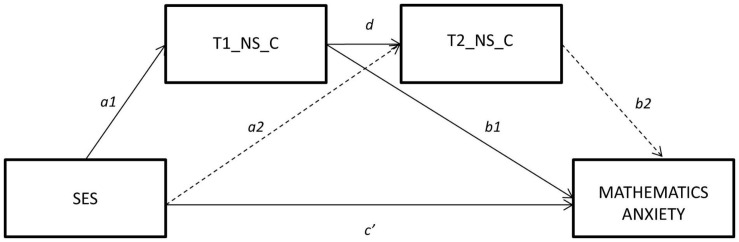
Serial Mediation Model for the Relationship Between socio-economic status and Mathematics Anxiety by longitudinal performance in non-symbolic numerical skills (Time 1 and Time 2). Dotted lines represent non-significant predictor pathways.

The data in this table are represented in Figures 1, 2. ** *p* < 0.01. *** *p* < 0.001.

#### Model 2

The second serial mediation model examined whether the SES-MA relationship was mediated by performance in longitudinal non-symbolic magnitude comparison (Figure 2). The results revealed that the total effect (combining direct and indirect effects) was significant (*c* = 0.12, *SE* = 0.02, *p* < 0.001). Like model, the total effect size_*cs*_ was 0.25, suggesting a large effect size. The total indirect effect of SES on MA was also significant, but the effect size was small (0.03). The specific indirect effects indicated that only the indirect path between SES and MA, mediated by early performance in non-symbolic comparisons in pre-school, was significant (*b* = 0.03, 95% CI = [0.01, 0.05]), while the path mediated by performance in non-symbolic comparisons 2 years later was no longer significant (95% CI included zero, *b* = −0.01, 95% CI = [−0.01, 0.00]).

## Discussion

The aim of the present study was to evaluate the relationship between SES, longitudinal performance in basic numerical skills, and MA in children at two time points (pre-school and second grade). Our hypotheses stated a direct effect of SES on MA and a mediating effect of basic numerical skills between SES and MA. To the best of our knowledge, there are no previous studies that have explored the role of both SES and numerical performance on MA. In the present study, two mediation models were conducted: Model 1 tested the mediating role of longitudinal performance in symbolic comparison tasks, and Model 2 examined the longitudinal performance in non-symbolic comparison tasks.

Results showed a direct effect of SES on MA, implying that the greater the level of vulnerability in pre-school and second grade students, the higher their MA levels. These results mirror those found in a number of studies that have investigated the effect of SES on MA among primary school (Adimora et al., 2015), secondary school (Organisation for Economic Co-operation and Development, 2013), and university students (Geyik, 2015). Although the above research is clear in showing that students from families with limited educational attainment and financial resources are at risk of developing MA (Rubinsten et al., 2018), this association has scarcely been studied in primary school children and had never been examined in children as young as 5 years.

It is worth mentioning, however, that not all previous studies have consistently found evidence of the SES-MA relationship (e.g., Mahigir et al., 2012; Ching, 2017). We believe that discrepancies in the literature are due to differences in approaches to measuring SES. For instance, Ching (2017) defined SES as the educational attainment of the mother, which is a rather simplistic measure. By contrast, all previous studies that have found evidence of a relationship between SES and MA have used compound measure of SES (Adimora et al., 2015; Geyik, 2015) which are believed to be more reliable than those which include few dimensions (Letourneau et al., 2011). Our study used a measure of SES that includes a set of economic, educational, occupational, and health criteria relating to subjects’ families, and our findings are in line with the results of those studies where similar measures were used. As such, we extend the evidence of the SES-MA relationship to 5-year-olds.

These results are particularly relevant for Chile, a Latin American country whose education system presents high levels of economic segregation, with high-SES students almost exclusively attending private schools and low-SES students attending public schools (Ortiz, 2015; Córdoba et al., 2017). The consequences of this segregation may be observed in the striking differences in numerical performance between children from high- and low-SES schools, with the former outperforming the latter (Sepúlveda et al., 2020). We showed that the effect of SES on MA is similar to that of SES on academic achievement. This is worrying given that MA levels tend to increase in severity as school level increases (Namkung et al., 2019) and with it, its relationship with mathematical performance (Lauer et al., 2018). The findings of the present study may provide greater cause for concern, in that Chilean children present higher levels of MA than those reported in studies involving the same educational year groups and the same measure of MA (Maloney et al., 2015; Ramirez et al., 2016; Van Mier et al., 2019), but conducted in contexts of greater socio-economic equality than that found in Chile. These findings, in line with the results of PISA (2013), suggest that contextual factors are relevant in the evaluation of MA, not only in adolescents, but also in young children.

Regarding the mediating role of basic numerical performance in the SES-MA relationship, our results confirmed our hypothesis, showing that the longitudinal development of symbolic and non-symbolic magnitude comparison skills does mediate this relationship. Our findings are in line with previous studies in adults suggesting a link between poor basic numerical skills and MA (Maloney et al., 2011; Núñez-Peña and Suárez-Pellicioni, 2014; Dietrich et al., 2015). Importantly, the present study shows that an early numerical deficit during pre-school, associated with differences in SES, contributes to performance differences in second grade and, in turn, to the appearance of unpleasant affective responses toward mathematics.

It should be noted that although the present work reveals a mediating role of early numerical skills in the SES-MA relationship, it also shows different contributions made by symbolic and non-symbolic skills over time. The results indicate that both symbolic and non-symbolic performance in pre-school are longitudinally linked to MA in second grade, which suggests that MA may be associated with a less precise comprehension of numerical magnitudes at early stages. Thus, in principle, our findings support a cognitive deficit model, in which MA emerges because of a deficit at a basic level (e.g., Maloney et al., 2011). However, the results also show that only performance in the symbolic comparison task continues to be related to MA in second grade. These findings coincide with evidence from studies in older children (Wang et al., 2015; Hart et al., 2016) and university students (Dietrich et al., 2015; Braham and Libertus, 2018) that failed to identify a relationship between non-symbolic comparison tasks and MA. The present findings suggest that performance in non-symbolic comparison tasks plays an important role in explaining MA at initial stages, but that its influence is no longer significant in second grade. The reason for this might be that non-symbolic numerical tasks have almost no resemblance to regular classroom activities associated with teaching or assessment, meaning that they could become disassociated from MA and cease to influence it at a very early stage. By contrast, as children’s formal educational experience in mathematics increases, MA becomes linked primarily to symbolic numerical tasks, even if these are not cognitively demanding. There is abundant evidence that basic symbolic numerical tasks are associated with MA in both children and adults (Maloney et al., 2011; Núñez-Peña and Suárez-Pellicioni, 2014), meaning that the present results are very much in line with the existing literature. Symbolic numerical tasks closely resemble classroom activities introduced by teachers at second grade (e.g., counting, cardinality), suggesting that children’s experience with a broad range of explicit numerical tasks, including those relating to assessment, may reinforce the early connection between performance in numerical comparison tasks and MA.

A novelty of the present study was the introduction of SES to explain MA and the extent to which its influence was mediated by performance in numerical skills. Until now, we suspected that the SES-MA relationship may be linked to the influence of instruction on the development of symbolic numerical skills (Jordan et al., 2010; LeFevre et al., 2010). Our findings confirm that SES does influence MA and that this influence is mediated by symbolic numerical skills, which are linked to classroom instruction. In the context of Chile’s highly segregated school system (Ortiz, 2015; Córdoba et al., 2017) in which disadvantaged students have fewer classroom learning opportunities, in part because they receive less instructional time than their higher-SES peers (Ponce and Strasser, 2019), the link between MA and performance in numerical skills becomes more prominent. We believe that low-quality teaching experience in low-SES schools is responsible for increasing both the direct effect of SES on MA and its mediating effect through performance in symbolic numerical skills. Chile’s particular context could benefit from the provision of enriched mathematical environments during pre-school education, targeting children from lower socio-economic backgrounds who have fewer opportunities for learning at home (Clements and Sarama, 2011). More specifically, early exposure to explicit instruction in symbolic and non-symbolic representations could mitigate initial differences derived from low SES, and favor the acquisition of fundamental concepts for an adequate later mathematical development (Chiatovich and Stipek, 2016; Scalise et al., 2017; Träff et al., 2018). The above might constitute an early preventive measure against MA.

## Conclusion, Limitations and Future Work

In the present study, we conclude that SES affects MA and that this is due in part to the effect of SES on the development of numerical learning in pre-school, which in turn has an impact on subsequent, more complex learning, ultimately leading to differences in MA. These results have important implications, not only for our understanding of the nature of MA, but also in terms of tackling and prevention of the condition. We therefore propose the implementation of numerical strategies based on simultaneous explicit instruction of symbolic and non-symbolic representations in pre-school as this could mitigate the initial differences derived from children’s socio-economic backgrounds and launch a positive cycle of strong early performance and low MA.

Given that children are nested in interrelated contexts (Bronfenbrenner and Morris, 2006), we consider that the implementation of numerical strategies in home learning environments is also relevant to prevent MA. It is important to notice, however, that parental involvement patterns and attitudes toward mathematics differ according to families’ socioeconomic background (e.g., Susperreguy and Davis-Kean, 2016), so MA may even increase in low-SES children (e.g., Jameson, 2014). For this reason, we suggest that numerical strategies at home should only be encouraged if schools provide advice to parents on how to interaction with their children during math homework (see Berkowitz et al., 2015, for a successful experience).

These results should be interpreted in the context of some limitations. First, this study was carried out in children who were transitioning from pre-school to elementary school, which is considered a highly stressful period (Parent et al., 2019). Children need to adapt fast to a series of changes such as rules and routines, physical space (a new arrangement of furniture within the classroom), and the extension of time devoted to disciplinary activities (Hirst et al., 2011). In this context, it is expected that SES would play an important role in the first manifestation of MA. Particularly, in Chile, children in primary school are exposed to formal teacher-directed academic instruction that differs from the balanced kindergarten methodology, which includes formal instruction on disciplinary aspects and the development of academic precursors, but without abandoning the playful learning activities that characterize pre-school education. Additionally, formal assessment is also incorporated in primary school, which is another possible source of MA (Caviola et al., 2017). Future studies should investigate whether the pattern of relationships between SES-performance-AM found in the present study is moderated by specific aspects of school transition. Finally, MA has traditionally been measured by self-reported questionaries and so was the case in the present study. These measures are limited in the sense that they cannot assess math anxiety as it unfolds before or during task performance. Hence, we encourage the use of online, more objective measures (i.e., physiological responses) in future studies, especially, when assessing young children who may find it difficult to identify and report specific emotions linked to anxiety.

## Data Availability Statement

The datasets generated for this study are available on request to the corresponding author.

## Ethics Statement

The studies involving human participants were reviewed and approved by Universidad Católica de la Santísima Concepción. Written informed consent to participate in this study was provided by the participants’ legal guardian/next of kin.

## Author Contributions

All authors contributed to the conceptualization and design of the study, to data analysis, and to the writing of the manuscript.

## Conflict of Interest

The authors declare that the research was conducted in the absence of any commercial or financial relationships that could be construed as a potential conflict of interest.

## Footnotes

1 The differences of means and distributions of the variables under study between the cases with missing values and without missing values did not differ significantly [*SVI*, *t* (611) = −1.99, *p* = 0.05; *T1_S_C*, *t* (611) = 0.742, *p* = 0.458; *T1_NS_C*, *t* (611) = 0.867, *p* = 0.386]. This was confirmed when conducting the Hawkins Test (*p* = 0.406), which suggested that there was insufficient evidence to reject MCAR.
